# Analysis of hepatitis non-treatment causes in a cohort of HCV and HCV/HIV infected patients

**DOI:** 10.7448/IAS.17.4.19645

**Published:** 2014-11-02

**Authors:** Karen Pereira, Ana Cláudia Miranda, Teresa Baptista, Susana Peres, Isabel Antunes, Fernando Ventura, Fernando Borges, Kamal Mansinho

**Affiliations:** Infectious Diseases, CHLO, E.P.E, Hospital de Egas Moniz, Lisboa, Portugal

## Abstract

**Introduction:**

The decision to start hepatitis C virus (HCV) treatment and its timing remains controversial. As new treatment regimens are approved, it is essential to identify patients eligible for each regimen in a timed and tailored approach. This study aims to identify the reasons to defer treatment of chronic hepatitis C infection in both HCV and HCV/HIV infected patients.

**Materials and Methods:**

Retrospective observational study of a cohort of HCV chronically infected patients with or without HIV infection, followed in an infectious disease clinic in Lisbon. Demographic, epidemiological, clinical, immunologic and virologic data were collected. Statistical analysis was performed with Microsoft Office^®^- Excel 2012. Kolmogorov-Smirnov, t-test, Chi-square and correlation analysis were performed for a significant p value<0.05.

**Results:**

The study included 669 patients, 225 patients infected with HCV (group A) and 444 patients co-infected with HCV/HIV (group B). The comparative analysis of those groups (A vs. B) showed: mean age was 49.4 years versus 46.9 (p<0.01), mean time since HCV diagnosis was 9.5 versus 14.6 years (p=0.558) both groups shared a male predominance and HCV acquisition due to intravenous drug use. Regarding genotype characterization, the predominant was 1a in both groups (p<0.01). Evaluation of IL28B polymorphism revealed CC 15.5% (A) versus 9.45% (B) (p<0.01). Group B mean TCD4 count was 585 cells/µL (mean percentage 27.1%). There was spontaneous viral clearance in 10.7% (A) versus 4.1% (B) (p<0.01). There were treated 52.0% (A) versus 32.2% (B) patients (p<0.01). For the untreated ones (107 – group A vs 270 – group B), no reason was identified for treatment deferral in 32.5% (A) versus 48.0% (B) patients. The most frequent reasons for deferring treatment were: withdrawal to follow-up (33.7%), active staging of disease (7.2%), alcohol abuse (6.0%) and advanced age (6.0%) in group A versus low TCD4 cell count (17.1%), loss to follow-up (7.5%), poor adherence (7.5%) and alcohol abuse (3.2%) in group B.

**Conclusions:**

One of the highlighted cause for treatment deferral in both mono and co-infected patients was withdrawal to follow-up. In co-infected patients, low TCD4 cell count and poor adherence, also gain prominence, suggesting that strategies to improve retention in care may be needed. Additionally, emergence of direct-acting antiviral agents is expected to reduce these determinants in starting treatment, namely reduce the impact of low TCD4 count in co-infected patients.

